# Male-specific age estimation based on Y-chromosomal DNA methylation

**DOI:** 10.18632/aging.202775

**Published:** 2021-03-11

**Authors:** Athina Vidaki, Diego Montiel González, Benjamin Planterose Jiménez, Manfred Kayser

**Affiliations:** 1 Department of Genetic Identification, Erasmus University Medical Center Rotterdam, Rotterdam 3000, CA, The Netherlands

**Keywords:** Y-chromosome, epigenetics, DNA methylation, epigenetic age prediction, machine learning

## Abstract

Although DNA methylation variation of autosomal CpGs provides robust age predictive biomarkers, no male-specific age predictor exists based on Y-CpGs yet. Since sex chromosomes play an important role in aging, a Y-chromosome-based age predictor would allow studying male-specific aging effects and would also be useful in forensics. Here, we used blood-based DNA methylation microarray data of 1,057 males from six cohorts aged 15-87 and identified 75 Y-CpGs with an interquartile range of ≥0.1. Of these, 22 and six were significantly hyper- and hypomethylated with age (p(cor)<0.05, Bonferroni), respectively. Amongst several machine learning algorithms, a model based on support vector machines with radial kernel performed best in male-specific age prediction. We achieved a mean absolute deviation (MAD) between true and predicted age of 7.54 years (cor=0.81, validation) when using all 75 Y-CpGs, and a MAD of 8.46 years (cor=0.73, validation) based on the most predictive 19 Y-CpGs. The accuracies of both age predictors did not worsen with increased age, in contrast to autosomal CpG-based age predictors that are known to predict age with reduced accuracy in the elderly. Overall, we introduce the first-of-its-kind male-specific epigenetic age predictor for future applications in aging research and forensics.

## INTRODUCTION

Epigenetic age estimation based on age-correlated DNA methylation has emerged as the most accurate and robust molecular estimator of biological age [1]. DNA methylation age (DNAm) based on 353 autosomal CpGs (Cytosine-phosphate-Guanine dinucleotides), identified from DNA methylation microarray data, represents the most widely used multi-tissue age predictor with high accuracy (error of ± 3.6 years across tissues) [1]. This so called Horvath clock was developed from microarray data of >8,000 individuals and 51 healthy tissues, and has also been tested for the effect of accelerated aging in disease, such as in obesity [2], HIV infection [3] and cancer [4]. Over the years, the success of epigenetic age predictors has been continued, targeting different tissues e.g. blood and buccal cells [5] and semen [6], particular age groups e.g. children [7], expanding from humans to other species e.g. chimpanzees [8] and mice [9], as well as utilizing multiple statistical approaches [10] and targeted laboratory methods, e.g. next-generation sequencing [11].

However, all currently available epigenetic age predictors are based on CpGs located on the autosomal chromosomes. Nevertheless, sex-specific differences in epigenetic mechanisms exist, including in the X-chromosome inactivation in women [12], sex-specific genome-wide DNA methylation patterns [13], sex-specific epigenetic regulation of gene expression [14], and also in sex-specific aging-related phenotypes and diseases [15]. Particularly, it has been reported that male, but not female, longevity advances as a result of rising male mortality; this leads to the mortality type-dependent sex gap in longevity between males and female being further broadened [16]. Furthermore, epigenetic mechanisms define the sex-specific stage for disease later in life [17]. More importantly, the sex chromosomes code for various epigenetic modifiers that are differentially expressed between the two sexes, which can potentially affect the autosome in a sex-specific way [18]. But despite the evident sex differences in some disease, for example cardiovascular diseases, epigenetic analysis on this topic so far is not always stratified by sex, indicating that sex-specific DNA methylation might still have to give us additional insights in such disease mechanisms [15]. All this evidence suggests for a putative role of sex chromosomes in human aging; therefore, age-associated epigenetic changes may also exist on the sex chromosomes, like the Y-chromosome.

Thus far, the Y-chromosome is used as a popular genetic tool in phylogeny and population history [19] as well as in forensics [20]. Recently on the epigenetics side, Zhang et al showed that the DNA methylation pattern on the Y-chromosome was stable among family members and haplogroups, as well as conservative during human male history [21]. The authors were able to identity haplogroup-specific Y-CpG methylation sites, which were both genotype-dependent [21]. On the other hand, current literature on the human Y-chromosome and aging is mostly limited to the (mosaic) loss of the entire Y-chromosome in blood and buccal cells in aging men [22, 23]. Only recently, age-dependent DNA methylation patterns on the Y-chromosome were explored for predicting all-cause mortality in elderly males [24]. Lund et al. investigated the age association of Y-linked DNA methylation (416 Y-CpGs in total) in four datasets of males (n=624 in total) [24]. They identified 219, 76, 40 and 169 Y-CpGs exhibiting age-dependent methylation patterns, with 7 being shared among all cohorts. Interestingly, the vast majority of age Y-CpGs were hypermethylated over age as shown by comparing the regression coefficients in cohorts with increasing mean age [24]. Despite these promising results, age-dependent DNA methylation patterns on the Y-chromosome have not yet been investigated in a large age range for the purpose of developing a male-specific age predictor. Having such a predictor would eventually be relevant for studying male-specific effects on ageing, improving autosomal-based age prediction and also for specialized forensic applications.

In the forensic context, the male perpetrator of a crime is often not identifiable with standard forensic DNA analysis based on short tandem repeats (STRs). When the police has no hits at the national DNA database and/or no suspect for a crime, predicting the physical characteristics of the unknown person via forensic DNA phenotyping (FDP) [25] may provide useful investigative leads. Among the phenotypes of interest, age is a distinct personal characteristic that influences the way a person appears; therefore, predicting age from crime scene DNA is a very useful piece of evidence to narrow down suspect pools. Existing autosomal CpG-based age predictors show great promise due to their great accuracy but are only applicable to single-source DNA samples, meaning to DNA samples that belong to a single biological donor. Current autosomal CpG-based age estimation in males, if coupled with an additional, independent Y-chromosome-based age predictor, would potentially lead to a more confident age estimation. Particularly in cases where we obtain mixed male-female DNA samples, as often obtained in physical or sexual assault cases, a Y CpG-based male-specific age predictor would also enable the prediction of the age of an unknown male perpetrator. As a result, such male-specific age predictor would also allow us to discriminate among close male relatives belonging to the same paternal lineage but are of different age, such as father vs son, who are indistinguishable in current forensic Y-chromosomal DNA analysis, because they typically share the same Y-DNA haplotype [20].

In this study, we aimed to investigate the age correlation of Y-chromosomal DNA methylation in a wider age range, which is expected to lead to new clues in sex-specific molecular processes of aging given that it has been systematically excluded in most autosomal CpG-based age DNA methylation studies. For this, we used publically available DNA methylation data obtained by the Illumina® Infinium® HumanMethylation450 Beadchip array in whole blood, as blood is one of the most commonly used medical/(public) health-related and forensically relevant body fluid. Hence, we aimed to develop a blood-based male-specific Y-CpG based age predictor that could be relevant not only to study male-specific aging in epidemiology or age-related diseases, but also in forensics for male donor-specific age prediction.

## RESULTS

### Age correlation of Y-CpGs in blood

**Figure 1:**
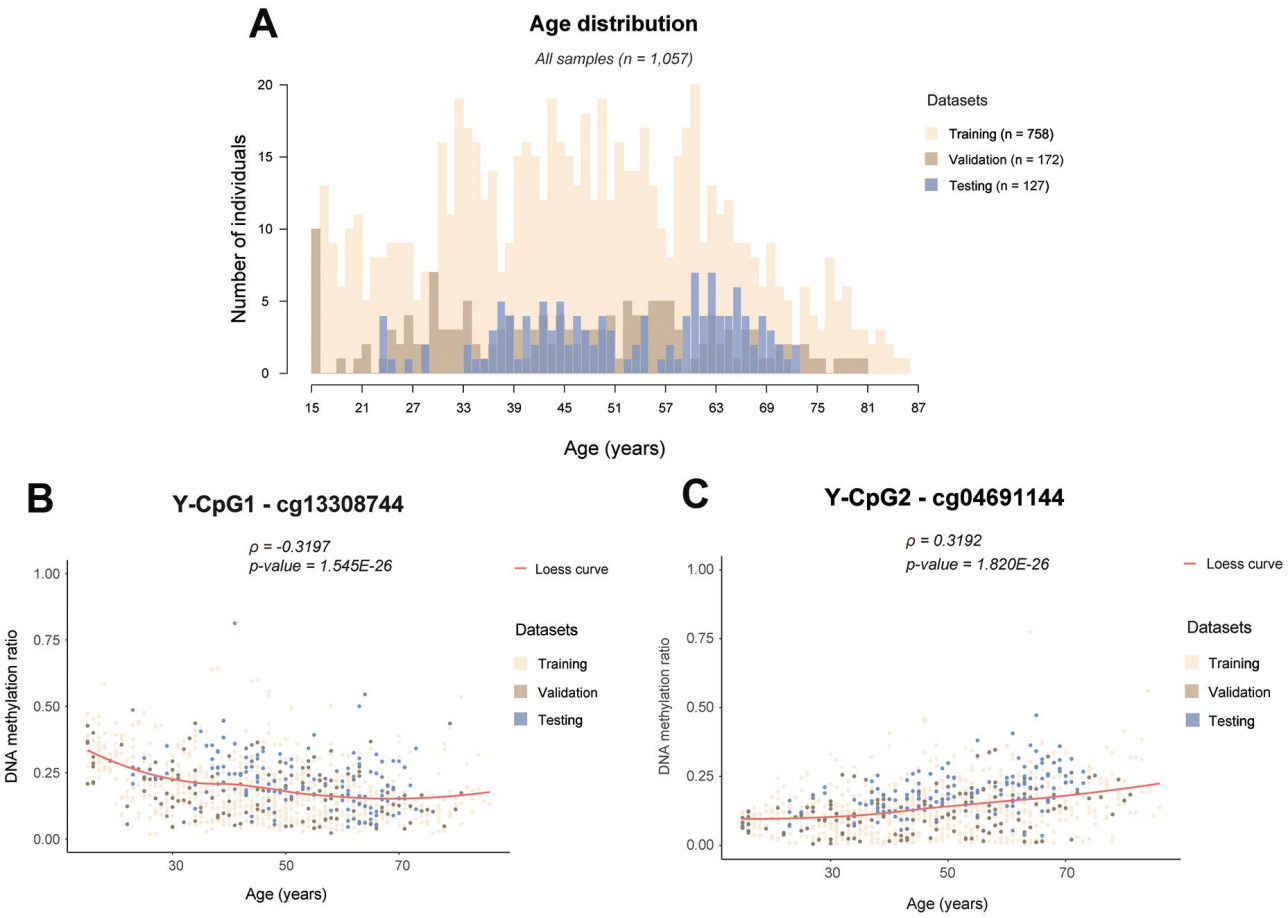
**Examples of age-associated Y-CpG methylation in blood.** (**A**) Histogram showing the age distribution in all samples colour-coded per training (n = 758), internal validation (n = 172) and external testing (n = 127) datasets, (**B**) DNA methylation levels of cg13308744 showing the strongest negative correlation with age (ρ = -0.3197, p-value = 1.545E-26), (**C**) DNA methylation levels of cg04691144 showing the strongest positive correlation with age (ρ = 0.3192, p- = 1.820E-26). ρ: Spearman correlation coefficient, Bonferroni threshold: α/n= 0.05/75 = 6.667E-4, Loess: locally estimating scatterplot smoothing curve.

We used publicly available Illumina® Infinium® HumanMethylation450 Beadchip microarray data that cover 416 Y-CpGs. We collected such data from six studies (Table 1) previously generated from blood of 1,057 healthily aging males of a wide age range (15-87 years old) (Figure 1A). These datasets were initially produced to investigate the correlation of autosomal DNA methylation with birth weight [26], aging [27], stress [28], allergic rhinitis [29] and insulin resistance [30], while their Y-chromosomal data remained entirely unexplored as of yet.

**Table 1:** Information on the six publicly available Illumina® Infinium® HumanMethylation450 BeadChip datasets used in this study.

***GEO dataset***	***No. of samples***	***No. of males***	***No. of samples following QC***	***Health status***	***Age range (years old)***	***Tissue***	***Used for***
*GSE100386*	46	24	24	Healthy/Rhinitis	21-61	Lymphocyte-enriched PBMCs	Training Validation
*GSE125105*	699	312	275	Healthy/depressed	18-79	Whole blood	
*GSE128235*	537	229	214	Healthy/depressed	20-79	Whole blood	
*GSE61496*	312	82	76	Healthy	30-74	Whole blood	
*GSE87571*	732	341	341	Healthy	15-87	Whole blood	
*GSE115278*	474	132	127	Healthy	23-73	Peripheral white blood cells	Testing

**Figure 2:**
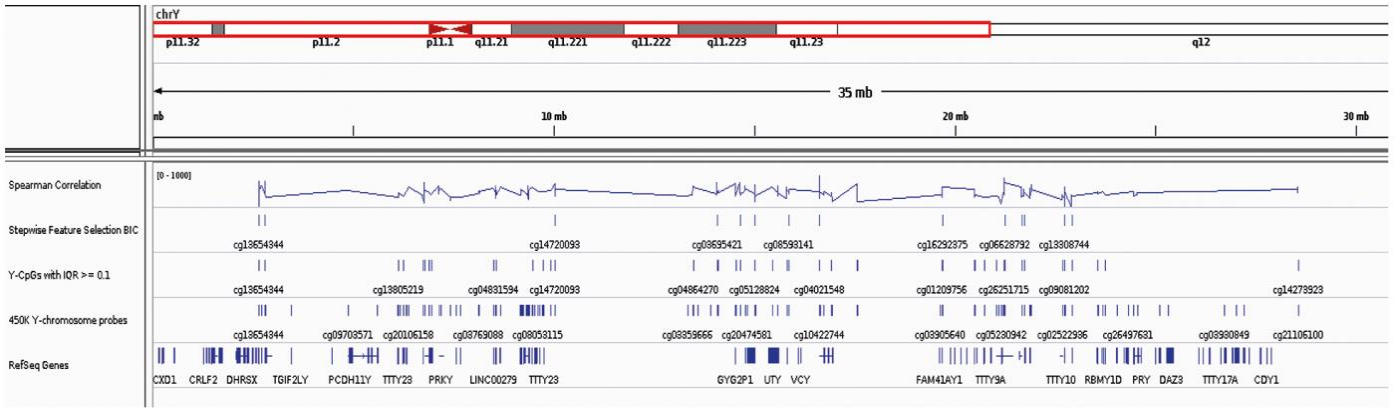
**IGV screenshot on the Y Chromosome including the location of all reference Y-genes, the 416 Y-CpGs included in the Illumina® Human Methylation450 BeadChip array and the 75 age-predictive Y-CpGs used in this study (with highlighted the 19 Y-CpGs that were further selected) as well as their Spearman correlation coefficients.**

Following strict quality control as described in the Methods, our initial marker pool consisted of a total of 268 (64%) of the 416 Y-CpGs covered by the microarray. We found that Y-CpGs are more variable compared to their autosomal counterparts (p-value = 2.64×10-6, Supplementary Figure 1). To enrich for Y-CpGs displaying biological variation rather than purely technical noise, we applied a strict empirical threshold of inter-quantile range (IQR) ≥ 0.1 that further reduced our marker pool to 75 male-specific Y-CpGs, scattered across the entire Y-chromosome (Supplementary Table 1). By computing a Spearman correlation coefficient that allows to measure correlation with age that follows non-linear monotonic relationships, we found that 22 Y-CpGs (29.3%) were hypermethylated and 6 Y-CpGs (8%) were hypomethylated with age (Supplementary Figure 2). Our results confirm a tendency of increased hypermethylation of Y-CpGs with age; however, their effect sizes tend to be small. Given that the relationship between DNA methylation change and age for these age-related Y-CpGs does not follow a linear trend, we did not calculate the percentage increase in methylation per unit age, as it is expected to vary with time. Nevertheless, when comparing the very young (< 20 years old) versus the elderly (> 70 years old), we observed a ~15% average decrease or increase in DNA methylation for our top age-related hypomethylated and hypermethylated Y-CpGs, respectively (Figure 1B, 1C). The computed Spearman correlation coefficients ranged between -0.3197 for cg13308744 showing the most significant negative age correlation (Figure 1B) and 0.3192 for cg04691144 showing the most significant positive age correlation (Figure 1C). In total, 28 Y-CpGs (37.3%) showed age correlation on the significance level of 5% and 23 Y-CpGs on the significance level of 1%. The correlation coefficients for all 75 CpGs are presented in the Supplementary Table 1 and their position on the Y-chromosome in Figure 2.

### Age prediction based on Y-CpGs in blood

Next, we implemented several supervised machine learning algorithms listed in Table 2 to find the best performing age prediction approach. For model building, we used five out of the six available datasets that were randomly split into an 80% model training set (n = 758) and a 20% model validation set (n = 172) by maintaining a homogenous and wide age distribution in both data subsets. The sixth original dataset (n = 127) was normalized separately and used as an independent external model testing dataset. Using multiple linear regression (MLR), we achieved a mean absolute deviation (MAD) between predicted and observed age of 10.45 years (ρ = 0.65) in the validation dataset and 9.31 years (ρ = 0.58) in the external testing dataset (Table 1). Similar MADs were obtained using other methods such as lasso, ridge and elastic net regression, all of which capture linear relationships only (Table 2). Regularization and built-in feature selection in the lasso and elastic net models (32 and 33 age-predictive Y-CpGs respectively, Supplementary Table 1) did not affect age prediction accuracy (Table 2). Additionally, we applied random forest regression (RFR), which delivered reduced MAD of 9.23 years (ρ = 0.80, validation dataset) when using all 75 Y-CpGs, and 9.63 years (ρ = 0.74, validation dataset) when using a sub-selection of the 19 best-predictive Y-CpGs (Table 2 and Supplementary Figure 3B). For all methods, similar and slightly improved MADs were obtained for the independent external testing dataset compared to the internal validation dataset (Table 2).

**Table 2:** Metrics of machine learning approaches applied for Y-based epigenetic age prediction.

**Regression model**		**Parameter(s)**	**No. of features(Y-CpGs)**	**Internal validation (n=172)^1^**					**External testing (n=127)^1^**			
				**MAD (years)**	**ρ**	**RMSE**	**R^2^**		**MAD (years)**	**ρ**	**RMSE**	**R^2^**
*Multiple Linear*		N/A	75	10.45	0.65	12.93	0.42		9.31	0.58	11.65	0.34
*Lasso*		α: 1	32*	10.71	0.65	12.99	0.42		9.19	0.58	11.08	0.34
*Ridge*		α: 0	75	10.67	0.66	12.88	0.43		8.76	0.60	10.70	0.36
*Elastic Net*		α: 0.5	33*	10.72	0.65	12.99	0.42		9.15	0.59	11.01	0.34
*Random Forest*		ntree: 500, mtry: 25, nodesize: 5	75	9.23	0.80	11.33	0.64		8.48	0.66	10.18	0.43
			19^§^	9.63	0.74	11.89	0.54		8.42	0.61	10.50	0.37
*Support Vector Machine (ε-type)*	*Linear kernel*	C: 1	75	10.69	0.60	13.88	0.36		10.63	0.53	13.03	0.28
	*Polynomial kernel*	C: 1, degree: 3, γ: 0.013	75	9.41	0.68	12.40	0.46		9.71	0.53	12.48	0.28
	*Sigmoid kernel*	C: 1, γ: 0.013	75	13.83	0.40	17.83	0.16		11.44	0.33	16.90	0.11
	*Radial kernel*	**C: 2, γ: 0.013**	**75**	7.53**	**0.81**	**10.15**	**0.653**		7.61**	**0.70**	**9.36**	**0.49**
		C: 2, γ: 0.052	19†	8.46	0.73	11.77	0.53		8.88	0.57	11.38	0.33

MAD: Mean Absolute Deviation, ρ: Pearson Correlation Coefficient, RMSE: Root Mean Square Error, N/A: Not Applicable.

α: Regularization parameter, ntree: Number of trees to grow, mtry: Number of variables randomly sampled as candidates at each split, nodesize: Minimum size of terminal nodes, C: Cost weight for penalizing the soft margin, degree: Number of degrees for the polynomial equation, γ: Controls the trade-off between error due to bias and variance in the model.

^1^All models were built based on our training set (n = 758).

*Based on α penalization, which shrinks coefficients towards zero.

^§^Based on Random Forest Cross-Validation for feature selection.

**(in bold) Best performing model.

^†^Based on stepwise-feed forward feature selection and Bayesian Information Criterion (BIC).

**Figure 3:**
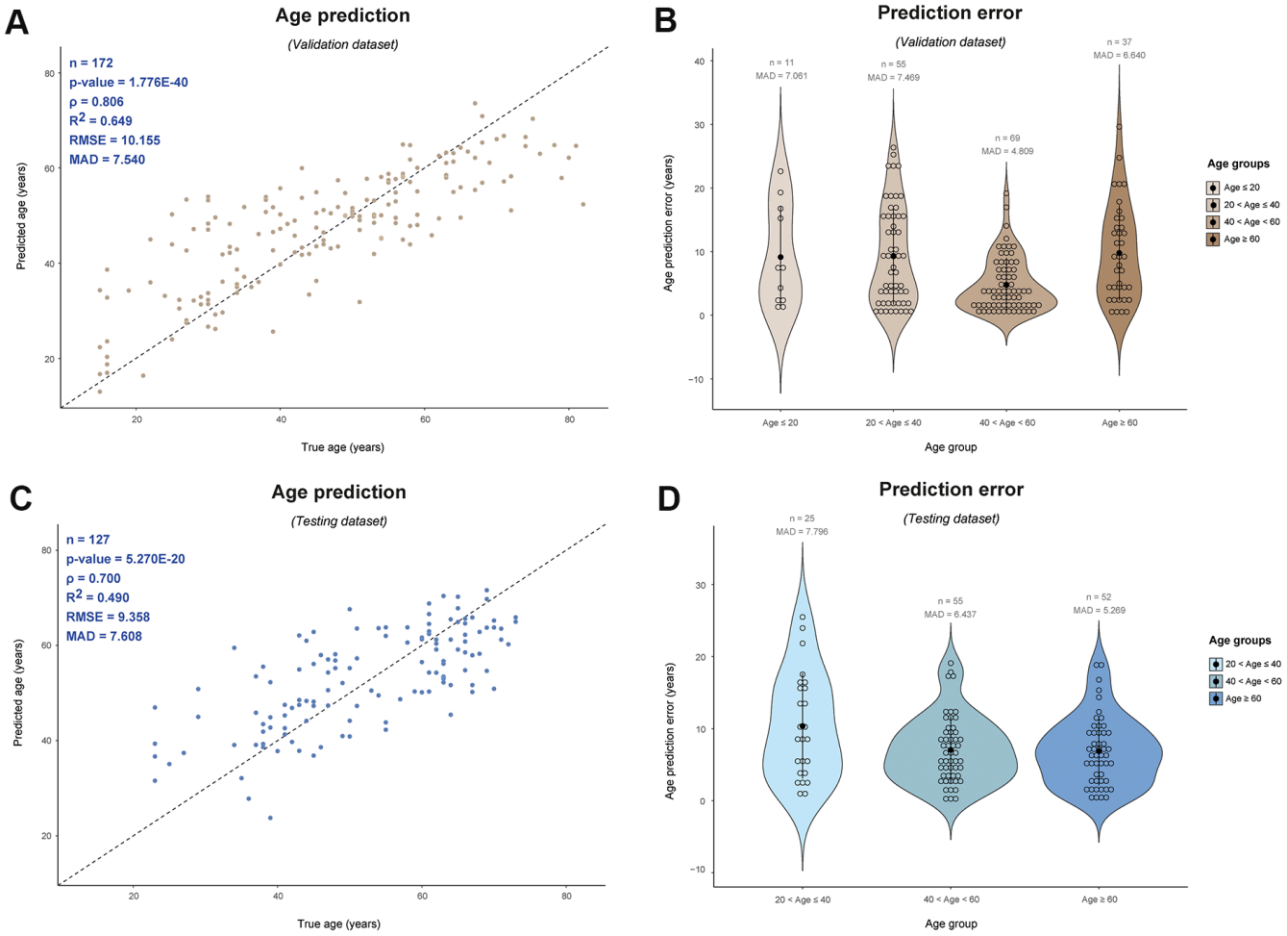
**Male-specific epigenetic age prediction in blood based on 75 Y-CpGs using support vector machine (radial kernel).** Validation dataset (n = 172): (**A**) Predicted vs. true age and (**B**) age prediction errors per age category; Testing dataset (n = 127): (**C**) Predicted vs. true age and (**D**) age prediction errors per age category. ρ: Spearman correlation coefficient, RMSE: root mean square error, MAD: mean absolute deviation.

In an attempt to further reduce the age prediction error by taking into account possible non-linear associations with age that we already observed for our top age Y-CpGs (Figure 1B), we applied support vector machine (SVM) using the ε-regression method implemented with different kernels. While the SVM linear model based on all 75 Y-CpGs resulted in similar MAD as the MLR model (10.69 years, ρ = 0.60, validation dataset), the SVM third-degree polynomial model improved age prediction by more than one year (9.41 years, ρ = 0.68, validation dataset) and the SVM radial model by more than three years (7,54 years, ρ = 0.81, validation dataset) (Table 2 and Figure 3A). The improved age prediction was also seen in the external testing dataset (7.61 years, ρ = 0.70) (Table 1 and Figure 3C). Even when including a stepwise-feed forward feature selection based on Bayesian Information Criterion (BIC) and reducing the Y-CpGs to 19 (11 shared with all other models, Supplementary Table 1 and Supplementary Figure 3A), the age prediction accuracy achieved with SVM remained better than in all non-SVM models (9.05 years, ρ = 0.71, validation dataset).

Additionally, there was an interesting observation concerning the age prediction accuracy across age groups, particularly for older individuals. In our 75-Y-CpG SVM radial model, we observed similar average prediction errors across age groups, meaning similar prediction accuracies in young (age ≤ 40) and elderly individuals (age ≥ 60) (Figure 3B). Particularly for the validation dataset, we obtained a MAD = 7.061 for the 11 individuals aged ≤ 20 years, MAD = 7.469 years for the 55 individuals aged between 20-40 years, MAD = 4.809 years for the 69 individuals aged 40-60 years, and finally, MAD = 6.640 years for the 37 individuals aged ≥ 60 years. Similarly in the testing dataset, we obtained a MAD = 7.796 years for the 25 individuals aged between 20-40 years, MAD = 6.437 years for the 55 individuals aged 40-60 years, while for the 52 older individuals aged ≥ 60 years the MAD was 5.269 years. Unfortunately, there were no individuals aged ≤ 20 years in the testing dataset.

### Comparison between our male-specific age estimator and the Horvath clock

**Figure 4:**
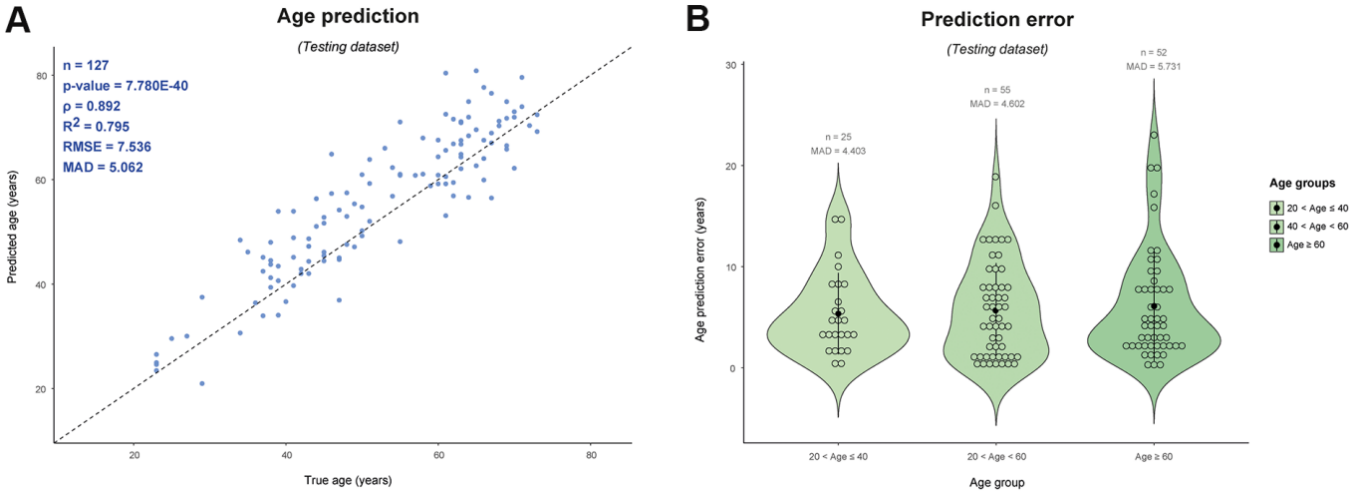
**Age prediction of male samples included in the testing set of this study (n = 127) using the publically available Horvath age predictor based on 353 autosomal CpGs** [1]**.** (**A**) Predicted *vs.* true age and (**B**) age prediction errors per age category. ρ: Spearman correlation coefficient, RMSE: root mean square error, MAD: mean absolute deviation.

As already mentioned, the Horvath clock represents the most widely used multi-tissue age predictor [1]. This highly accurate and robust age clock is based on 353 autosomal CpGs identified out of a pool of >450,000 CpGs included in the Illumina® 450K microarray and analysed with >8,000 samples, compared to our male-specific age estimator that is based on a smaller market set (75 Y-CpGs) identified out of a smaller marker pool (only 416 Y-CpGs) and analysed on a much smaller sample size (n = 1,057). We applied the Horvath clock on the very same samples used for our Y-based age estimator’s independent model testing and obtained a MAD of 5.06 years, which is almost two years larger than the reported by Horvath in whole blood (error of ± 3.7 years, testing dataset) [1] and less than three years smaller than the one obtained in our 75-Y-CpG SVM radial model (MAD = 7.61 years). In contrast to our model (Figure 3D), the prediction error variance based on the Horvath model slightly increased for individuals >60 years old; MAD of 5.73 years for age >60, compared to 4.40 and 4.60 years obtained for the other two age groups (Figure 4B).

### Functional annotation of the top age-correlated Y-CpGs

Our selected 19 Y-CpGs are scattered across the entire Y-chromosome (Supplementary Figure 3). The vast majority of them (18 out of 19) are located within Y-CpG islands and within Y-genes (12 out of 19) (File S1). These include a set of 10 genes, such as EIF1AY, DDX3Y, ZFY, TTTY14 and NLGN4Y. Mutations in and differential expression of these Y-chromosome genes, such as the DDXY3 gene, have been linked with male infertility and reduction of germ cell numbers [31, 32], which are age related processes, also in other tissues such as sperm [33]. Our finding of age-correlated CpGs existing in the rest of the Y-genes likely has an underlying biological reason, which should be investigated in future studies. Since the current literature on these genes in limited so far, we cannot exclude that they might be indirectly linked with aging.

Additionally, although excluding Y-CpG probes is a standard practice in epigenome-wide association studies (EWAS), we also checked the selected 19 Y-CpGs of the SVM radial model for possible reported associations with age-related traits or diseases in the EWAS atlas database [34]. Notably, ~5% lower methylation of cg00121626 is associated with the autoimmune disease, primary Sjögren's syndrome [35], while ~5% higher methylation of cg03767353 is associated with the Kabuki syndrome, caused by mutations in histone-modifying enzymes [36]. Lastly, 21% higher methylation of cg13654344 has been observed in prostate tumours [37].

## DISCUSSION

The main purpose of our study has a proof-of-principle nature, showing for the first time that CpGs on the Y-chromosome have the potential to be used for age estimation in males of a wide range of reproductive age. Previously, and in line with its DNA sequence variation, Y-CpG DNA methylation has been shown to be evolutionarily conserved, with stable DNA methylation patterns on the Y-chromosome reported among family members and haplogroups [21]. The authors of one of the only couple existing studies on Y-chromosomal DNA methylation also found two haplogroup-specific, both genotype-dependent, CpGs [21].

In our study, Y-CpGs seem more variable compared to the autosomal ones, which could at least be partly due to noisier DNA methylation detection. Technically, this could be explained by the single-copy nature of the Y-chromosome in contrast to the two copies of autosomal chromosomes resulting in half the possible signal, or by underlying biological reasons, such as reduced specificity of the designed probes [38]. But even though there are variable Y-CpGs, their observed association between DNA methylation levels and age is weak (ranging from -0.3197 to 0.3192), compared to what we are used to so far for autosomal age-CpGs. From the total 268 Y-CpGs used in this study, only 75 CpGs (27.99%) passed our variability threshold (IQR ≥0.1).

Our results confirm a tendency of increased hypermethylation of Y-CpGs with age, also reported by Lund et al., the only existing study exploring age correlation of Y-chromosomal DNA methylation with mortality in elderly males [24]. In comparison with the seven age-associated Y-CpGs reported by Lund et al., three Y-CpGs (cg03055837, cg00311963 and cg06636270) were removed from our analysis as cross-reactive based on lists reported by previous studies [38, 39]. This could mean that these Y-CpGs bind in multiple regions of the genome, therefore resulting in non-specific DNA methylation signal. Another three Y-CpGs (cg14180491, cg01707559, and cg18188392) were excluded from our predictive analysis following the IQR threshold (<0.1). Therefore, only one Y-CpG (cg00679624) was overlapping between our Y-CpG marker list and that of Lund et al. [24]. Lastly, if we look at the functional annotation, two out of the 10 genes reported in our study (Y-linked Neuroligin, NLGN4Y and DEAD-box helicase 3, DDXY3) were also reported by Lund et al. who also listed two other Y-linked testis-specific transcripts (TTTY20 and TTTY23) but not ours (TTTY14), strengthening the validity of our results on the age-correlated Y-CpGs.

Our Y-chromosome-based results are also similar with the ones obtained for other sex chromosome – the X-chromosome. In brief, from the total of 10,096 X-CpGs included in the study by Li et al. sex-specific X-linked DNA methylation changes over age later in life was recently identified at 123, 293 and 55 significant CpG sites in males, females and both sexes, respectively [40]. X-CpGs that are highly methylated in both sexes, similarly to the Y-CpGs, also tend to get hypermethylated even further with age. These results could indicate that the sex chromosomes undergo differential methylation changes during aging in comparison with the ones on the autosomal chromosomes [40].

Moving forward to prediction, regardless the low age correlation of our variable Y-CpGs which seemingly follow non-linear relationships, they still contain sufficient information that can be utilized in age prediction modelling. As expected, the selected 19 Y-CpGs in the SVM radial model included the Y-CpGs with the strongest positive/negative age correlation (Supplementary Table 1). The obtained MAD values of ~7-8 years are not as high as we are used to for autosomal-based age predictors based on similar number of CpG markers, but this is mainly driven by the smaller effect sizes and the weaker age correlations we observed for Y-CpGs. In the validation of our Y-CpG age predictor based on the 75 variable Y-CpGs (IQR ≥ 0.1) we obtained MAD values of 7.061, 7.469, 4.809, 6.640 years for individuals aged ≤ 20, 20-40, 40-60, and ≥ 60 years, respectively. The better age prediction accuracy in the 40-60 category can at least partly be explained by the bigger sample size in this age group. Similarly in the independent testing, we obtained MAD values of 7.796, 6.437, 5.269 years for individuals aged 20-40, 40-60, and ≥ 60 years. Unfortunately, there are no individuals in this dataset that fall in the first category (aged ≤ 20 years) to allow us strongly comment on the very young age category, but from both validation and testing, it seems that the age estimation accuracy in older individuals (> 40 years) is better than the younger ones (< 40 years). Similarly to above, less accurate age prediction in the 20-40 category can also at least partly be explained by the smaller sample size in this age group. But despite that, our model can still accurately distinguish between young adults and old individuals. This is particularly important for the potential forensic application where our Y age estimator will be used for differentiating close male relatives belonging to the same paternal lineage but are of different age, such as grandfather vs father vs son. All males of the same paternal lineage are expected to be indistinguishable with currently practiced Y-STR profiling, so a Y-based age predictor, without prediction accuracy bias towards specific age groups that these individuals might belong to, is a promising identification approach and could have additional investigative value when constructing the paternal branch of a pedigree.

Furthermore, we were interested to compare the performance of our Y-based age estimator with one of the most popular autosomal-based age clocks, the Horvath clock [1]. While a comparison between the two age predictors might not be considered totally fair given their differences, including the larger size of their training dataset, the >1000 times larger initial set of DNA methylation markers (CpGs), and the inclusion of markers across independent chromosomes, we were interested to see if an age estimator based on the Y-chromosome would behave similarly with one based on autosomal CpGs like the Horvath clock, which are known to underperform in older individuals [41, 42]. Despite the ‘unfair’ advantages of the Horvath clock, the results were promising for our male-specific age estimator. Using the same independent testing dataset, the MAD of predicted age using the Horvath clock was less than three years smaller than the one obtained in our 75-Y-CpG SVM radial model. We envision that with the use of a larger dataset as publically methylation microarray datasets becoming available as well as the use of Y-CpGs with stronger age correlation that still need to be identified, the performance of an age predictor based on the Y-chromosome will become comparable with the Horvath and other autosomal CpG-based age predictors.

For this proof-of-concept study we focused our analysis in whole blood, as it is one of the most commonly biological material collected in the clinic, research laboratory, or at the crime scene, such as in physical assault. Furthermore, there is currently a large depository of genome-wide DNA methylation data in the publically available domain. As a result, new age predictors based on the Y-chromosome of white blood cells can easily be compared with existing, thoroughly investigated autosomal-based predictors, such as the Horvath clock [1]. Additionally to blood, sperm could also be an interesting body fluid in ageing research due to its haploid nature and involvement in embryogenesis, but also due to its relevance in a wide range of civil (paternal), legal and criminal cases, such in sexual assault. Unfortunately, while there is a high number of datasets in whole blood, suitable data for non-blood tissues, such as sperm that could be relevant in sexual assault cases, are not of sufficient quantity or quality to conduct analysis of high power. For example, existing data in small numbers use a different type of platform used [43], do not include age information or include only elderly males (>70 years old, [44]). Nevertheless, we expect that the collection of large genome-wide DNA methylation data in sperm (such as by Jenkins et al, [6, 45]) will raise soon in the coming, ‘open-access’ era. This will allow us to expand our investigation to Y-chromosome-based age prediction in sperm; however, the constant production of sperm and the age-associated decreased sperm counts for males above 41 years of age, p = 0.023, ref) should be taken into account. For instance, men above 50 years have been reported to be 6.15 times more likely to present lower DNA amount in their semen compared to males aged 21-30 years [46], which subsequently then affects the process of DNA methylation detection.

Finally, while the Illumina® DNA methylation microarray data in blood exist in large numbers, that makes us able to achieve a high age predictive power, it is possible as a result, that their experimental procedures will vary greatly. This is expected to lead to a considerable DNA methylation variation, which should be taken into account in data analysis. To account for such methodological variation, we selected datasets with raw data available to enable harmonization via pooling and single pipeline normalization. However, for our independence testing, we performed a separate normalization of the data, mimicking the scenario of researchers applying our free, online Y-CpG-based age predictor (Availability information included in the Materials and Methods section). Also, Illumina technologies (27K, 450K, and currently EPIC) can analyse only a very small portion of the Y-chromosome methylome. Particularly, the Illumina® 450K platform contains the very small set of 416 Y-CpG markers out of the total of 217,906 existing Y-CpGs [47]). Novel tailored technologies that will allow Y chromosome-wide DNA methylation are required for expanding our analysis to more potentially biologically interesting Y-CpG DNA methylation in the future.

## CONCLUSIONS

In conclusion, we found age correlation of available Y-chromosomal CpGs in blood as well as built and validated the first-of-its-kind male-specific epigenetic age predictor for blood. This Y-chromosome-based age predictor is made available for future applications including in male-specific aging research as well as in more specialized areas of male-specific age prediction, such as age estimation in forensic applications. Future investigation of the Y-CpG markers included in the microarrays in other non-blood tissues such as sperm is expected to widen not only our knowledge in age-associated Y-CpG methylation but also practical forensic applications. Furthermore, future investigation of the entire Y-chromosome via a non-array methodology, such as whole genome bisulfite sequencing, is expected to result in the discovery of more age-correlated Y-CpGs, which shall be investigated for their age predictive value in addition to those presented here.

## MATERIALS AND METHODS

### DNA methylation datasets

Illumina® Infinium® HumanMethylation450 BeadChip array data from a total of 1,057 blood samples of male individuals aged between 15 and 87 years old were collected from six genome-wide DNA methylation studies, the raw data of which (IDAT files) had been made publically available via the Gene Expression Omnibus (GEO) database. Given that we targeted the Y chromosome for male-specific age prediction, we included exclusively male samples in this study. Samples were also carefully collected so that there was a broad age distribution (Figure 1A). We included only healthy individuals or individuals suffering from diseases like depression or rhinitis that are not expected to display strong effects on ageing. In particular, the GEO datasets we used are: GSE100386 [29], GSE125105 and GSE128235 [28], GSE61496 [26], GSE87571 [27] and GSE115278 [30]. More detailed information can be found in Table 1.

### Quality control (QC)

The entire analysis based on the Illumina® 450K methylation data was performed using R v3.5.2 [48], including quality control (QC), pre-processing and modelling. We implemented a QC workflow using the QCinfo function included in the ENmix R package [49] following default parameters (detPthre = 10-6, nbthre = 3, samplethre = 0.05, CpGthre = 0.05 and outlier = TRUE). Firstly, in the training and validation dataset, we discarded a total of 52 low-quality samples and 20,281 low-quality probes. In the testing dataset, we did not discard any sample but 7,555 low-quality probes. Additionally, we filtered out probes containing single-nucleotide polymorphisms (SNPs) in their sequence/CpG site/single-base extension site (n = 24,874), cross-reactive probes (n = 30,973) and probes associated to the X-Chromosome (n = 11,232). Altogether, we removed 73,699 and 62,811 probes from the two datasets, respectively. For both datasets we predicted the sex of our samples as implemented using the function *getSex* in the *minfi* v1.28.4 R package [50], which predicts sex based on the median values of measurements on both sex chromosomes. As a result of this analysis and to our surprise, we predicted five samples as females in the testing dataset (GSM3173076, GSM3173100, GSM3173105, GSM3173188, GSM3173434), which we excluded from subsequent analysis. Finally, regarding the data from GSE61496 (Danish twin study), we randomly selected one of each twin pair and excluded replicates.

### Data pre-processing and normalization

With respect to preprocessing, we firstly employed the function *ENmix::preprocessENmix*, in order to correct for background noise based on out-of-band (oob) probes and for dye bias via the REgression on Logarithm of Internal Control probes (RELIC) correction method [51]. Secondly, the function *ENmix::norm.quantile* was employed to quantile-normalize on separate Infinium type I/II probes and separate M/U (methylated/unmethylated) intensity channels. Finally, the function *ENmix::bmiq.mc* was used as a wrapper of the Beta Mixture Quantile dilation (BMIQ) method [52], which additionally corrects for Infinium type I/II probe bias. Samples used for model training and validation were normalized separately from the samples used for the independent model testing.

### Y-CpG sites

Only following QC and normalization that excluded cross-reactive, low-quality and SNP-containing Y-CpG probes, we retrieved the methylation values of a total of 268 (Y-CpGs, out of the 416 included in the Illumina^®^ 450K platform. To assess the hypothesis that Y-CpG probes are more variable compared to the autosomal ones, we compared the IQR distribution between autosomal and Y-chromosome probes using an one-sided Mann-Whitney U-test, which does not assume normality (Supplementary Figure 1). We then decided to filter out low-variation Y-CpGs presenting an IQR lower than an empirical, strict cut-off of 0.1. We tested each of the 75 Y-CpGs for significance in Spearman’s correlation employing the function *cor.test*. p-values were adjusted with Bonferroni multiple testing correction (α/n= 0.05/75 = 6.667E-4). In the end, we ended up with 75 Y-CpGs, annotated based on the IlluminaHumanMethylation450kanno.ilmn12.hg19 data (Supplementary Table 1). Additionally, Y-CpG probe positions on the Y Chromosome were visualized in Integrative Genomics Viewer (IGV) (Figure 2).

### Model building and testing

Based on five out of the six GEO datasets included in the study (n = 930, Table 1), and in order to choose the best performing age prediction approach, we implemented several supervised machine learning algorithms (Table 2) with *in house* R scripts. A hold-out cross-validation was included with an 80-20 % split between training (n = 758) and validation (n = 172) datasets, respectively. In order to maintain a homogenous and wide age distribution, we split randomly between age bins. For model building we used the age as response variable and the 75 Y-CpGs as independent variables. For model testing, we applied an external independent dataset (GSE115278, n = 127) that was normalized separately.

In our study various models were constructed. Firstly, for Ordinary Least Squares (OLS) for MLR, we used the *lm()* function from the standard R stats R package. Secondly, we applied shrinkage methods including (a) Ridge Regression, which penalizes the sum of squared coefficients (L2 penalty), (b) Lasso Regression, which penalizes the sum of absolute values of coefficients (L1 penalty) and (c) Elastic Net Regression, which is a convex combination of Ridge and Lasso. For these methods we trained our models using the function *cv.glmnet* in the *glmnet* R package [53]. Large coefficients are penalized by a λ (lambda). To define the best λ we used the Mean Square Error (MSE) as type of measure, 5 as the number of folds during Cross-validation (CV) and an α (alpha) of 1, 0 and 0.5, for Ridge, Lasso, Elastic net, respectively. Additionally, for RFR we employed the *randomForest* R package [54] using 500 as the number of trees (ntree), 25 as the number of random variables for each split (mtry) and 5 as the minimal size of terminal nodes (nodesize). Finally, for SVM we employed the *eps-regression* (ε) method using the *e1071* R package [55], that includes different kernels, such as linear, polynomial (degree: 3), sigmoid and radial basis function. To avoid over-fitting we implemented a grid-search for hyper-parameter optimization next to hold-out CV and we assessed using our internal validation dataset. Each kernel included a cost parameter (c) of 1 or 2, and default gamma (γ) of 0.013 (1/n, n = 75 CpGs). Overall, to assess machine learning performance, we made use of standard performance measures for regression, such as MAD, coefficient of determination (R-squared, R^2^), Root Mean Square Error (RMSE) and Pearson correlation coefficient (ρ) between true and predicted age.

### Feature selection

Towards an effort to reduce the number of features, we additionally applied forward stepwise regression as model refining using the BIC. This method uses different combinations of input parameters by adding one feature (Y-CpG) at a time until exhaustion. Furthermore, Lasso and Elastic Net Regression that both apply L1 penalization allow to limit the size of coefficients, which might also causes some of them towards zero. This also led to partial models using a sub-selection of Y-CpGs. We also included a feature selection based on Random Forest CV [56], which uses the feature importance function based on Gini impurity. Each decision tree in the Random Forest tries to minimize the residual sum of squares (RSS) when splitting each node, which resulted also in the selection of 19 (but different) features using the function *rfcv* with five CV (Supplementary Table 1).

### Horvath age clock

We also predicted DNA methylation (DNAm) age of our testing samples (n = 127) using the popular 353 autosomal CpG-based Horvath age clock [1], using the *agep* function included in the wateRmelon v1.28.0 R package [57].

### Resource

All (pre- and post-normalized) data used in this study and the SVM radial models based on 75 and 19 Y-CpGs have been released to the public domain under an MIT license at GitHub (https://github.com/genid/Y-CpG/) and at the Zenodo digital object identifier-assigning repository (https://doi.org/10.5281/zenodo.4304487).

### Data availability

The data that support the findings of this study are openly available in GEO at https://www.ncbi.nlm.nih.gov/geo/, with reference numbers GSE100386, GSE125105, GSE128235, GSE61496, GSE87571 and GSE115278.

## Supplementary Material

Supplementary Figures

Supplementary Table 1
