# Supplementary material for: Male-specific age estimation based on Y-chromosomal DNA methylation
Source: Aging (Albany NY). 2021 Mar 11;13(5):6442–58. doi: 10.18632/aging.202775 (PMC7993701; doi:10.18632/aging.202775)
Supplement: Supplementary Figures [file aging-13-202775-s001.pdf]

SUPPLEMENTARY FIGURES

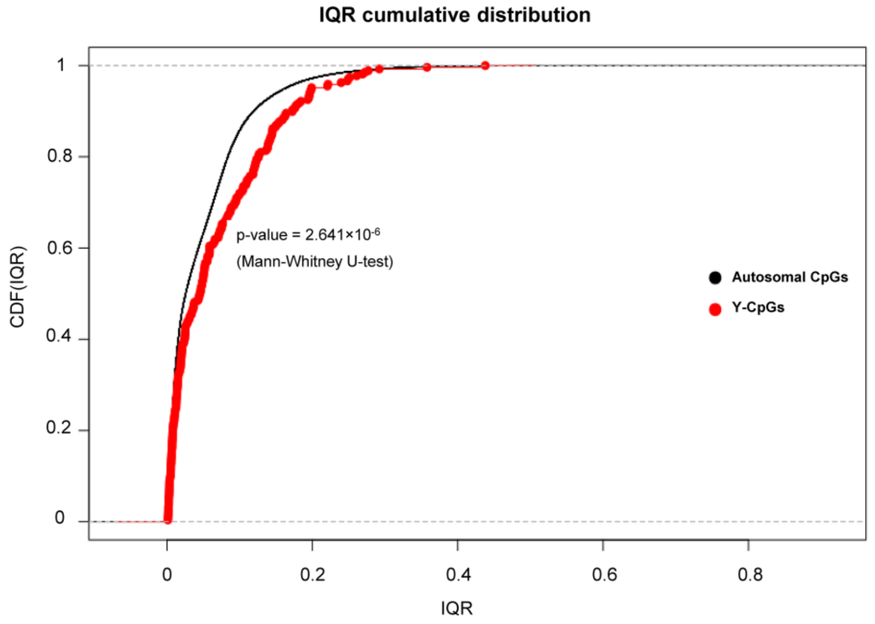

Supplementary Figure 1. Cumulative density distribution (CDF) of inter-quantile range (IQR) for autosomal (black and Y-chromosome (red) probes.

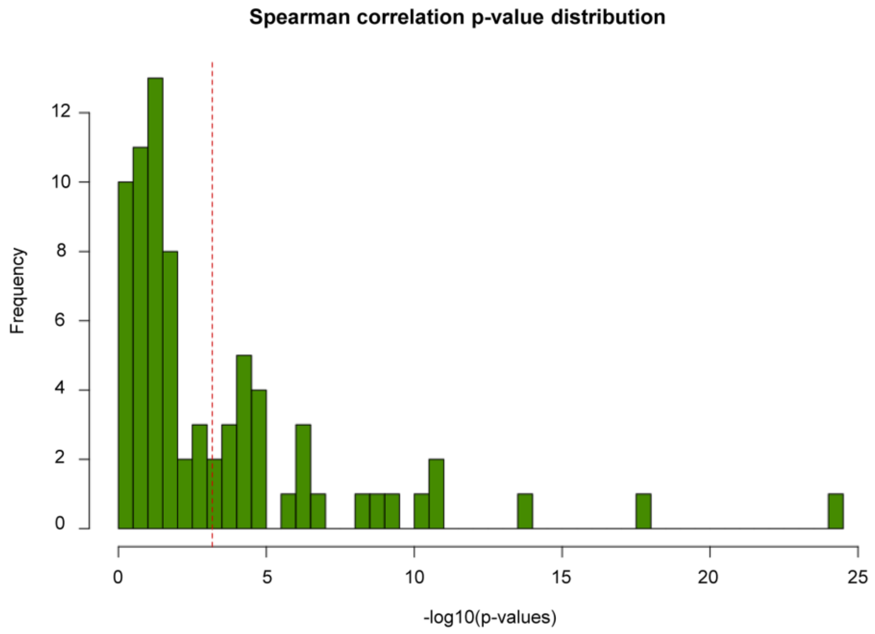

Supplementary Figure 2. Distribution of –log10(p-values) based on Spearman correlation test for all 75 Y-CpGs following the IQR threshold of ≥0.1. The dotted red line represents the $-\log_{10}$ of the Bonferroni-corrected degree of significance (α/n) =0.05/75.

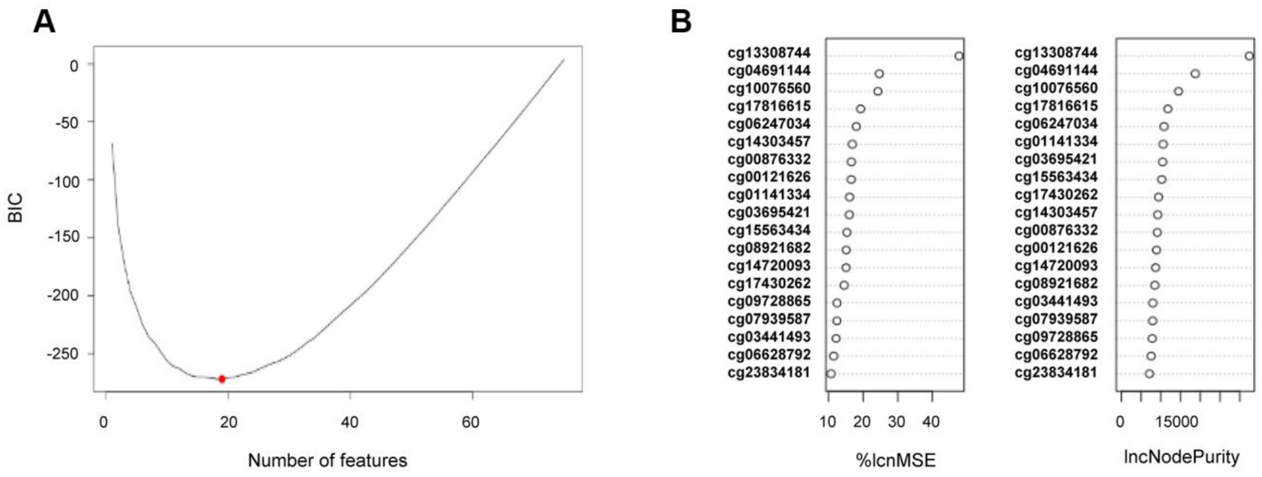

Supplementary Figure 3. Feature selection of age-predictive Y-CpGs. (**A**) Stepwise-feed forward feature selection with Bayesian Information Criterion (BIC), (**B**) Feature selection based on the Random Forest Regression model.
